# Fungal diversity in the gut microbiome of young South African children

**DOI:** 10.1186/s12866-022-02615-w

**Published:** 2022-08-17

**Authors:** K Nel Van Zyl, A. C. Whitelaw, A. C. Hesseling, J. A. Seddon, A-M Demers, M. Newton-Foot

**Affiliations:** 1 Division of Medical Microbiology, Department of Pathology, Stellenbosch University, Stellenbosch, South Africa; 2 National Health Laboratory Service, Tygerberg Hospital, Cape Town, South Africa; 3 African Microbiome Institute, Stellenbosch University, Stellenbosch, South Africa; 4 Desmond Tutu TB Centre, Department of Paediatrics and Child Health, Stellenbosch University, Stellenbosch, South Africa; 5 Department of Infectious Diseases, Imperial College London, London, UK; 6 Service de Microbiologie, Département Clinique de Médecine de Laboratoire, Centre Hospitalier Universitaire Sainte-Justine, Montréal, Canada

**Keywords:** Mycobiota, Gut fungi, Children, ITS, Microbiome

## Abstract

**Background:**

The fungal microbiome, or mycobiome, is a poorly described component of the gut ecosystem and little is known about its structure and development in children. In South Africa, there have been no culture-independent evaluations of the child gut mycobiota. This study aimed to characterise the gut mycobiota and explore the relationships between fungi and bacteria in the gut microbiome of children from Cape Town communities.

**Methods:**

Stool samples were collected from children enrolled in the TB-CHAMP clinical trial. Internal transcribed spacer 1 (ITS1) gene sequencing was performed on a total of 115 stool samples using the Illumina MiSeq platform. Differences in fungal diversity and composition in relation to demographic, clinical, and environmental factors were investigated, and correlations between fungi and previously described bacterial populations in the same samples were described.

**Results:**

Taxa from the genera *Candida* and *Saccharomyces* were detected in all participants. Differential abundance analysis showed that *Candida* spp. were significantly more abundant in children younger than 2 years compared to older children. The gut mycobiota was less diverse than the bacterial microbiota of the same participants, consistent with the findings of other human microbiome studies. The variation in richness and evenness of fungi was substantial, even between individuals of the same age. There was significant association between vitamin A supplementation and higher fungal alpha diversity (*p* = 0.047), and girls were shown to have lower fungal alpha diversity (*p* = 0.003). Co-occurrence between several bacterial taxa and *Candida albicans* was observed.

**Conclusions:**

The dominant fungal taxa in our study population were similar to those reported in other paediatric studies; however, it remains difficult to identify the true core gut mycobiota due to the challenges set by the low abundance of gut fungi and the lack of true gut colonising species. The connection between the microbiota, vitamin A supplementation, and growth and immunity warrants exploration, especially in populations at risk for micronutrient deficiencies. While we were able to provide insight into the gut mycobiota of young South African children, further functional studies are necessary to explain the role of the mycobiota and the correlations between bacteria and fungi in human health.

**Supplementary Information:**

The online version contains supplementary material available at 10.1186/s12866-022-02615-w.

## Background

Fungi are poorly studied organisms of the gut ecosystem, and it is estimated that they only represent 0.001 – 0.1% of the organisms in the gastrointestinal tract. Nonetheless, this still equates to about a billion organisms that have substantially larger genomes than that of prokaryotes in the gut [1]. Fungi therefore have the potential to contribute a substantial amount of genetic material and metabolic products to the microbiome. These fungi and their genetic products were first referred to as the mycobiome by Ghannoum and colleagues in 2010 [2].

The mycobiota is a known reservoir for potential pathogens, also known as pathobionts, and there is increasing evidence linking the gut mycobiota to intestinal inflammatory diseases [3–6]. The disruption of fungal communities by the overgrowth of certain pathobionts has also been linked to disease in early life [7], including type 1 diabetes [6], diarrhoea-associated intestinal candidiasis [8] and focal intestinal perforation in neonates [9]. However, despite the recent rise in interest, the gut mycobiota has not been as widely explored as the bacterial microbiota. The composition and development of healthy gut mycobiota during early life has not yet been fully established and the transient nature of the mycobiota makes this difficult to do.

The Human Microbiome Project found that the gut mycobiota of healthy adult volunteers was dominated by the phylum Ascomycota and, to a lesser extent, Basidiomycota [10]. *Candida* spp. (Ascomycota), *Saccharomyces* spp. (Ascomycota), and *Malassezia* spp. (Basidiomycota) were the most commonly detected fungal genera. When compared with the bacterial microbiota in the same individuals, the mycobiota were found to be less diverse, and more variable over time [10]. Only a limited number of studies have investigated the infant mycobiota, and thus far only in developed nations. Studies conducted in Europe and the United States of America have identified *Candida* and *Debaryomyces* (Ascomycota) as the most abundant genera [11, 12], with *Candida* being especially prevalent in preterm infants [13]. *Saccharomyces cerevisiae* was found to be more abundant after the first year of life [12, 14].

The mycobiota is affected by factors such as physiology and lifestyle, the immune system and to a lesser extent, host genotype. Interactions with the bacterial microbiome may also influence the mycobiota [15]. Some of the more notable examples of fungal-bacterial correlation, summarized by Cui and colleagues [15], include the occurrence of *Mycobacterium* superinfection and aspergillosis, and the suppression of fungal growth by *Pseudomonas aeruginosa* in cystic fibrosis patients. The Human Microbiome Project described significant strong negative correlations between *Penicillium* and *Faecalibacterium*, and *Saccharomyces* and Lachnospiraceae in the healthy human gut [10]. The interactions between bacteria and fungi in the gut could influence human health and it is therefore important to describe the mycobiota and bacteriota together, as well as any significant correlations found between bacteria and fungi in the population.

In South Africa, the mycobiota has mainly been studied in wine-making; however, a sequencing-based study was recently performed in healthy adult volunteers [16]. This study reported that genera belonging to the Ascomycota, *Pichia*, *Candida*, and *Cladosporium*, dominated the mycobiota and that diet and geographical location played the most important roles in differentiating the gut fungi between those from urban and rural communities. There is a distinct lack of mycobiota data from low resource settings, and especially in paediatric populations. We set out to characterise the diversity and community structure of the gut mycobiota of young South African children, investigate the relationships between clinical, environmental, and socio-economic factors and the mycobiota, and identify potential relationships between bacteria and fungi in this population.

## Results

### Participant demographics

From November 2017 to July 2019, baseline stool samples were collected from 116 of 218 children enrolled in the Cape Town arm of the TB-CHAMP trial. DNA was extracted successfully from 115/116 samples; one sample and matched participant data were therefore excluded from further analysis. The children included in the study had a median age of 32 months (interquartile range [IQR]:15—43), 52.2% were boys and only one child was HIV-positive. The full set of participant demographics and exposures to different clinical and environmental factors that were investigated, as outlined in the methods section, have been published as part of a study describing the bacterial microbiota in the same samples, based on 16S rRNA gene sequencing [17].

### Fungal microbiota composition

Fungi were detected in all participant stool samples, but during paired-end analysis it was noted that *Saccharomyces* was not detected in the mock community control as expected, nor in any of the samples. This was attributed to the unusually long (480 bp) internal transcribed spacer 1 (ITS1) region in *Saccharomyces* spp., which did not allow overlap and merge of the paired-end reads (2 × 200 bp). Forward read only analysis successfully identified *Saccharomyces* in the mock community and was able to detect more taxa than paired-end analysis (Supplementary data and Table S1); therefore, further analysis was performed using the forward reads only. For the forward reads, 11,351,965 sequencing reads were produced; after clean-up and chimaera removal 9,180,236 remained and were used to produce feature data.

**Fig. 1:**
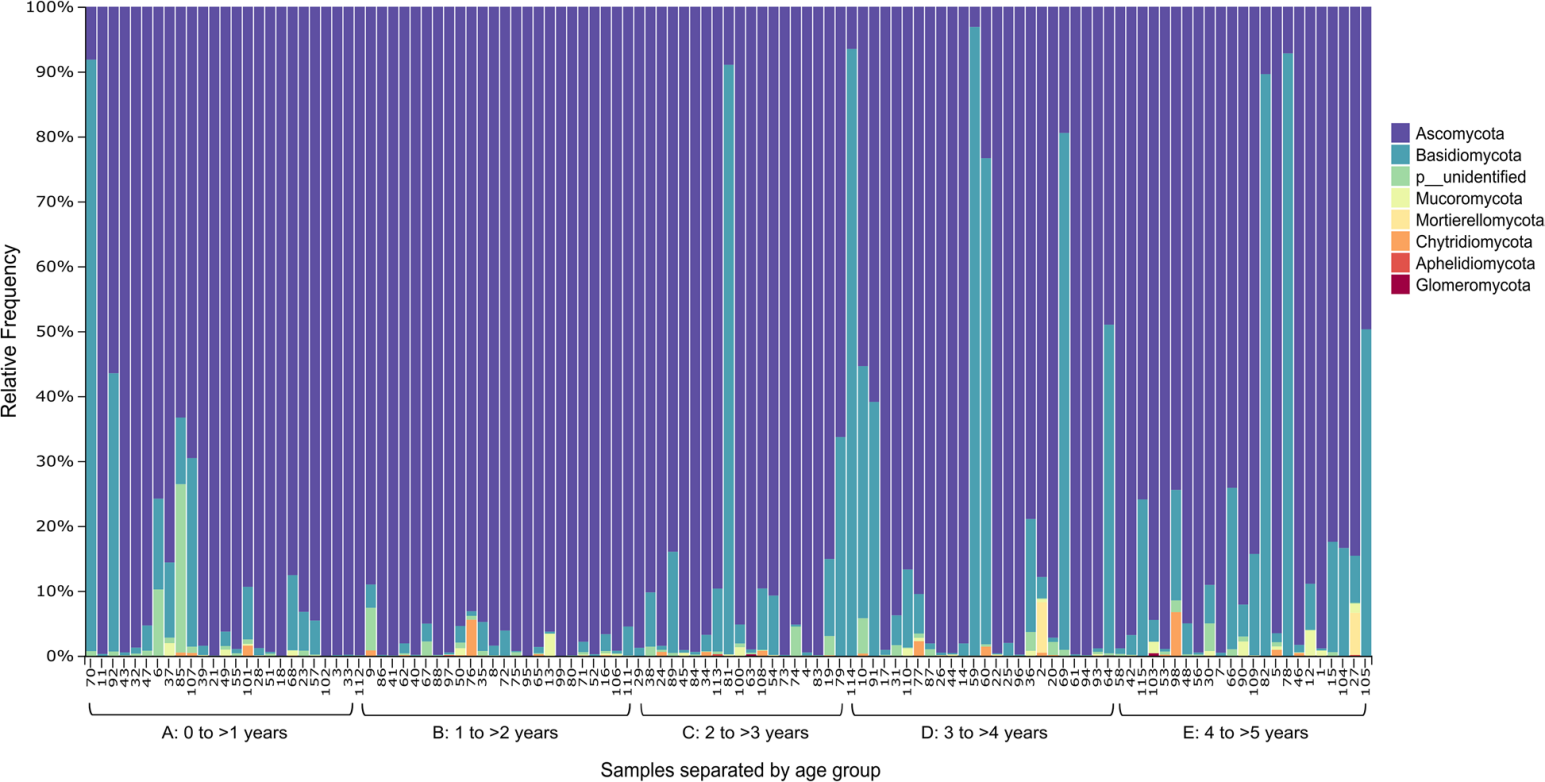
Taxonomic profiles of participant samples at phylum level. Each vertical bar represents a single participant sample. Features unassigned at phylum level were filtered prior to visualization. The brackets on the x-axis show the different age groups (**A** to **E**) and samples within each group are ordered by increasing age

The phylum Ascomycota was present in the highest abundance (94.8%), followed by Basidiomycota (4.8%) (Fig. 1). The remainder of the detected phyla were present in low abundance (< 1%) and/or detected in less than 5 samples.

**Fig. 2:**
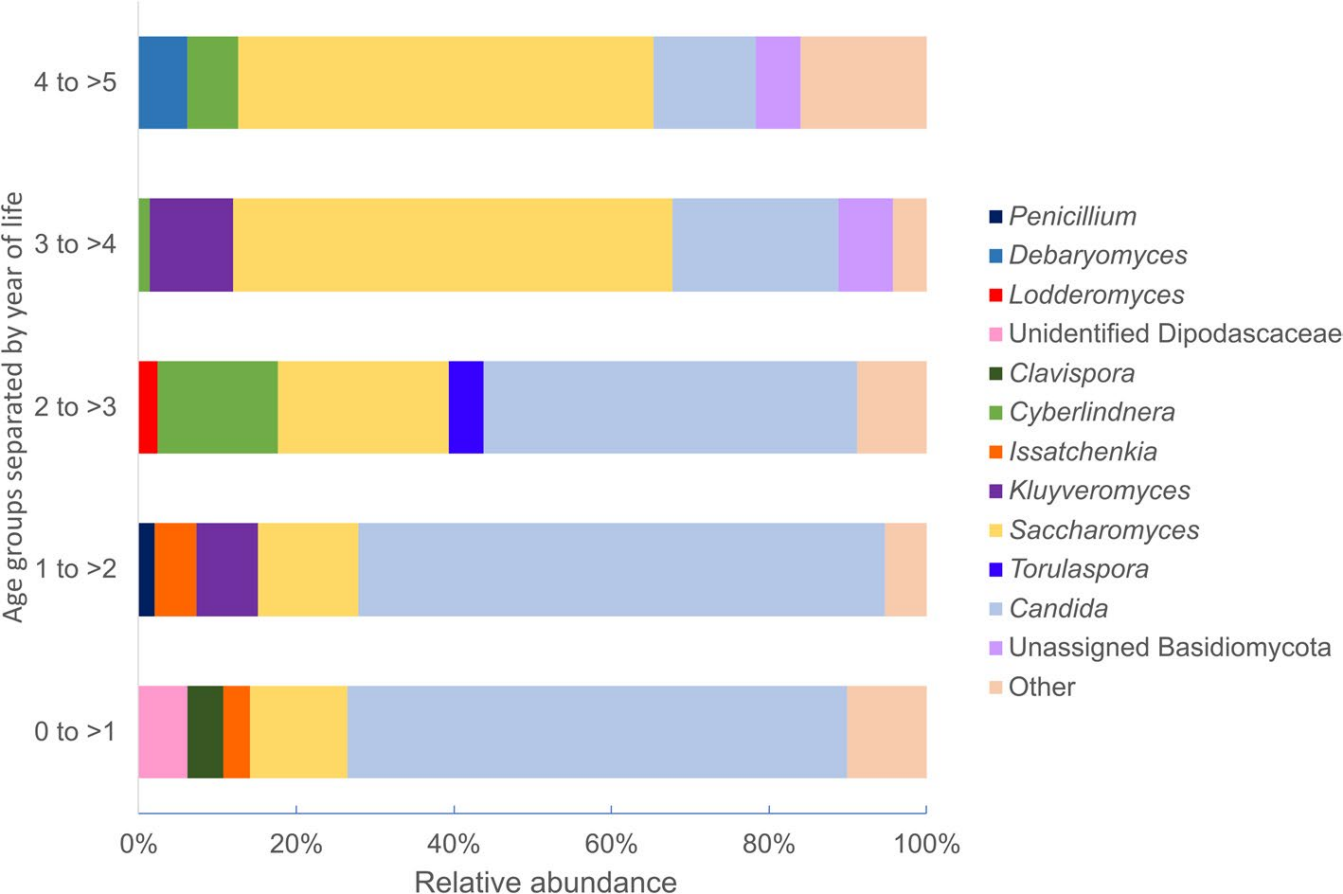
The relative abundance of the top five taxa in different age groups. Taxa that could not be classified to phylum level and features appearing in less than 5 samples were filtered prior to analysis

After filtering rare features that appeared in less than 5 samples, 67 populations could be identified, 53 to genus level, 6 to family level, 3 order level, one to class level and 4 to only phylum level. Despite the variety of fungi detected, the gut mycobiota was shown to be dominated by two genera, *Candida* and *Saccharomyces*, regardless of age group (Fig. 2).

At genus level, differential abundance analysis showed that *Candida* was significantly more abundant in children under 2 years, whereas *Cyberlindnera* was significantly more abundant in children over 2 years. While *Saccharomyces* was also shown to be more abundant in children older than 3 years, and *Debaryomyces* in children older than 4 years, the ANCOM results were not significant between any of the age groups. No other significant changes in abundance were noted for the tested factors.

### Alpha and beta diversity

**Fig. 3:**
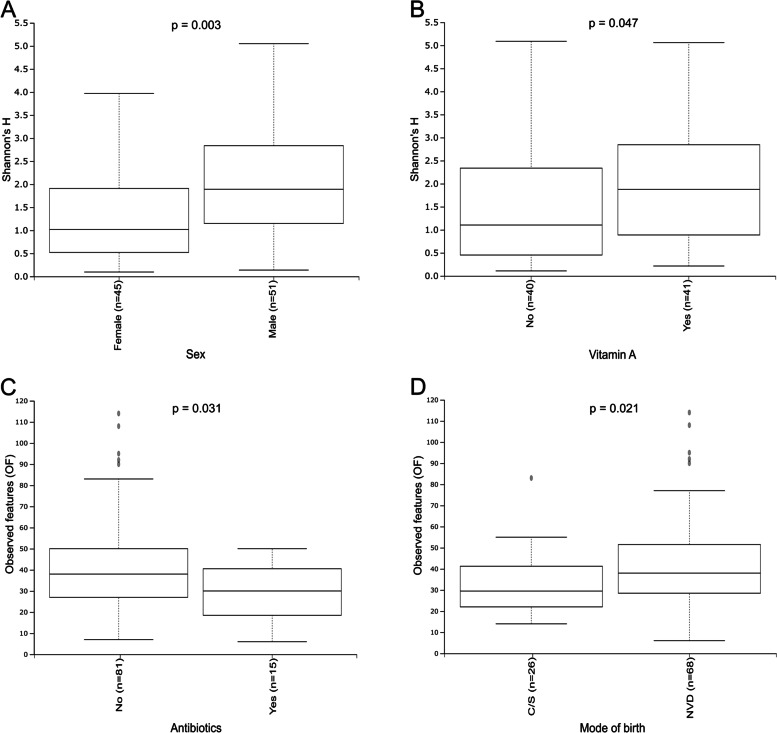
Significant differences in fungal alpha diversity as measured by **A** Shannon’s H alpha diversity and B observed features, based on Kruskal–Wallis testing (*p* < 0.05). A: Sex (Shannon). **B** Vitamin A supplementation in children > 6 months (Shannon). **C** Antibiotic exposure < 2 weeks before sample collection (OF). **D** Mode of birth (OF)

Nineteen samples were excluded from diversity analyses after rarefying to a sequence count depth of 5262. At this level of rarefaction, the Good’s coverage for all samples were > 0.97. For analysis of demographic, clinical and environmental factors, samples with no data were automatically excluded. As shown by Kruskal–Wallis testing, only four of the clinical, environmental, and socio-economic factors had significant associations with fungal alpha diversity based on Shannon’s H or observed features (OF) (Fig. 3, Supplementary Tables S2 and S3). Girls were found to have significantly lower alpha diversity (*p* = 0.003) and those older than 6 months who had received vitamin A supplementation in the preceding six months, had higher alpha diversity (*p* = 0.047). However, when considering only OF, these factors were not significant (girls vs boys, *p* = 0.06; vitamin A supplementation, *p* = 0.61). Children who had received antibiotic treatment in the two weeks before sample collection had lower richness as measured by OF (*p* = 0.03), but this was not significant with Shannon’s H (*p* = 0.069). Children who had been born with normal vaginal delivery had more observed features than those born by Caesarean section (*p* = 0.02).

**Fig. 4:**
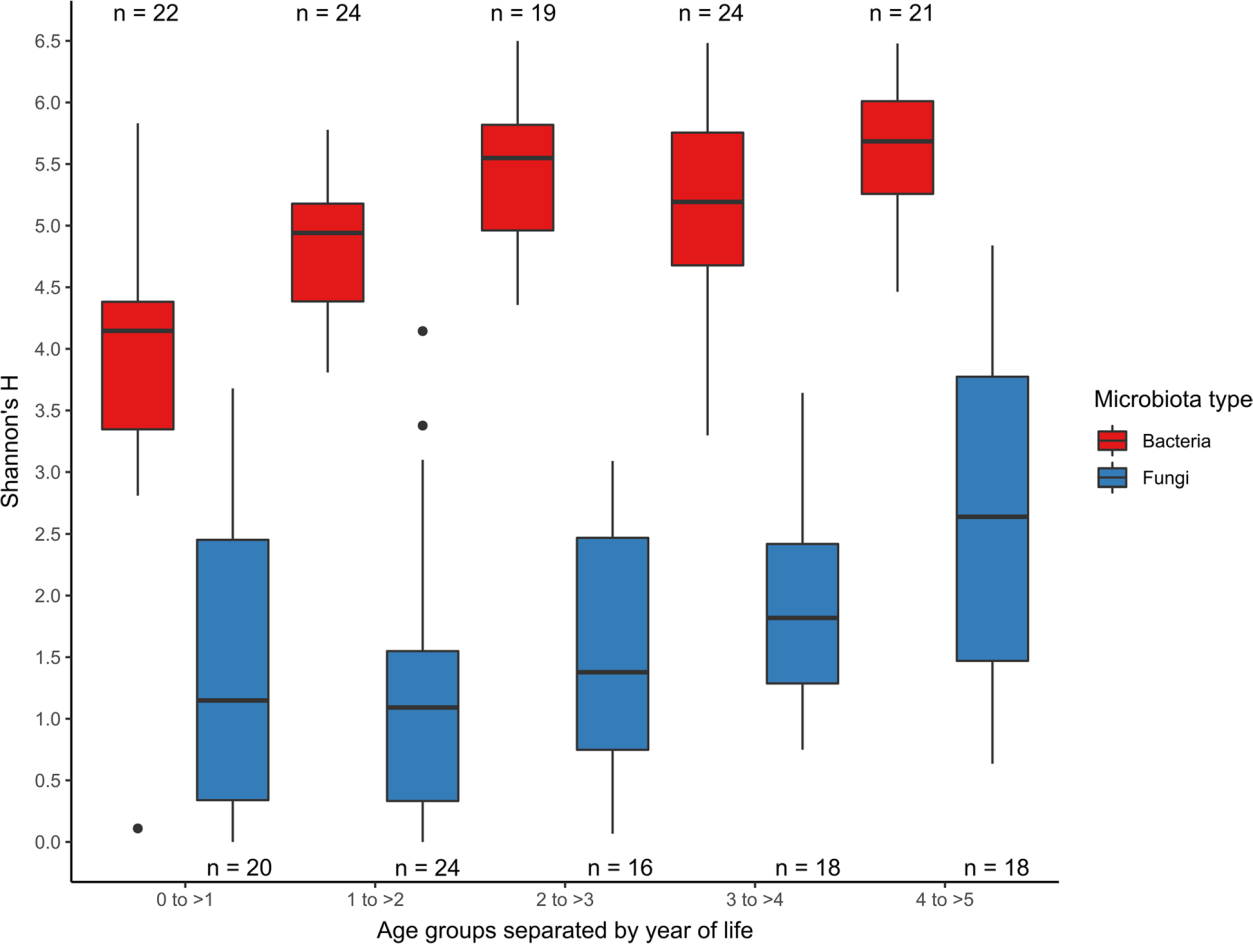
A comparison of bacterial and fungal diversity in children as measured by Shannon’s H alpha diversity. After rarefaction, 5 samples were excluded from bacterial diversity analysis (*n* = 110), and 19 samples from the fungal analysis (*n* = 96)

The alpha diversity of fungi in the gut was consistently lower and had a larger range than that of their bacterial counterparts in all the age groups, based on Shannon’s H metric (Fig. 4). Bacterial diversity was significantly lower in the younger age groups, and stabilised in children from the age of 3 years, whereas the differences in fungal alpha diversity between age groups were not significant when considering either Shannon’s H or OF (Supplementary Table S4).

Of the tested factors, only age contributed to fungal community dissimilarity, using the Bray–Curtis metric, Jaccard distance and PERMANOVA for significance evaluation. For both metrics, the two youngest age groups (0 to < 1 and 1 to < 2) were significantly dissimilar (adjusted *p* value < 0.05) to the two oldest age groups (3 to < 4 and 4 to < 5). R^2^ values as reported by the adonis plug-in are shown in Supplementary Table S5.

### Correlations between bacteria and fungi in the gut

**Fig. 5:**
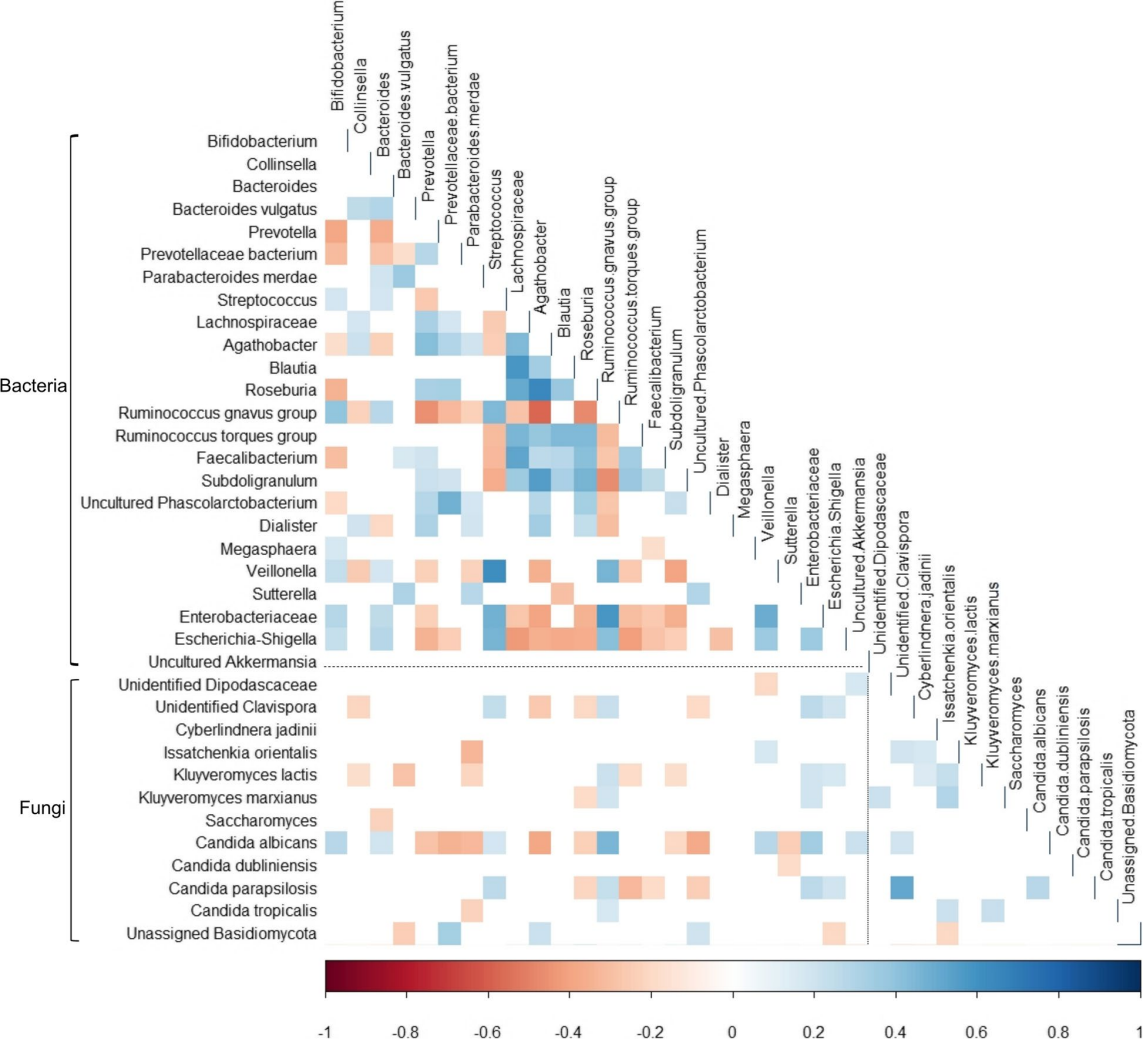
Correlations between bacterial and fungal taxa in the gut. Red represents negative correlations and blue represents positive correlations. Only significant correlations (*p* < 0.05) have been coloured

Both positive and negative bacterial-fungal correlations were observed in this population, although none were strong (r >  ± 0.7) (Fig. 5). The *Ruminococcus gnavus* group and members of the Enterobacteriaceae family exhibited positive correlations with almost all the represented fungal taxa, the strongest of which was between the *R. gnavus* group and *Candida albicans* (*r* = 0.451). Features belonging to *C. albicans* exhibited the most correlations with bacterial taxa, which is not surprising as it was the most abundant fungal species in this population. The strongest negative bacterial-fungal correlation was between *Agathobacter* and *C. albicans* (*r* = -0.388). Fungal-fungal correlations were mainly represented by co-occurrence, of which the association between *Candida parapsilosis* and the unidentified *Clavispora* spp. was the strongest (*r* = 0.518). The strongest positive correlations observed in this population were between the bacterial genera *Agathobacter* and *Roseburia* (*r* = 0.645), and *Streptococcus* and *Veillonella* (*r* = 0.613).

## Discussion

This study investigated the mycobiota composition in young South African children enrolled in the TB-CHAMP clinical trial. The richness and evenness of mycobiota in the gut varied substantially, and age was the main factor contributing to differences in fungal diversity.

Even though fungi are present in very low abundance in the gut, fungal taxa were detected in all the stool samples tested in the present study, and *Candida* and *Saccharomyces* dominated the gut mycobiota. Culture-independent studies previously performed in children identified *Candida* and *Debaryomyces* as the most abundant genera [12, 14], and similarly to the present study, *Saccharomyces* has been found at a higher relative abundance after infancy [12, 14, 18]. Many other taxa were detected in the present study, but they were sparsely distributed between samples, and > 80% of the mycobiota abundance in each age group was comprised of only 5 taxa. This sparseness was also seen in a cohort of healthy adults, where individual fungal operational taxonomic units (OTUs) were only detected in 3% of participants on average [19]. The majority of the most commonly detected genera in our study were similar to those found in higher abundance in adults, including *Candida*, *Saccharomyces*, *Aspergillus*, *Malassezia*, *Cyberlindnera*, *Penicillium*, and *Cladosporium* [10, 19]. A recent review summarised evidence that suggests that mode of birth and feeding practises both influence mycobiota composition in early life [20]. In this study, it was noted that children born by Caesarean section had less observed features than those born by normal vaginal delivery; however, no differentially abundant features were identified, and the fungal gut communities were not significantly dissimilar between these groups. Another study has suggested that fungi may be acquired from various sources such as the mother’s breastmilk, parental skin or even the hospital or home environments, due to their ubiquitous nature [14].

Fungal alpha diversity was significantly lower in girls, in contrast to other reported findings. A study in neonates found that the diversity and structure of fungi in the gut were unaffected by sex, whereas another study that included both adults and children found that the gut mycobiota in females had increased richness and abundance [11, 13]. This disparity in diversity between girls and boys was not detected in the bacterial microbiota of our study population [17]. The lower degree of diversity in girls may be a consequence of different diets, play activities and socialisation, medication use or immunological responses. We were not able to evaluate this due to limitations in our clinical data, but this important and novel finding requires further evaluation. To our knowledge, the association of vitamin A supplementation in children older than six months with increased fungal diversity has not been reported elsewhere and it is unclear what the underlying cause may be. Evidence suggests that deficiencies in micronutrients such as vitamin A may disrupt microbiota development, but published works focus on the bacterial microbiota [21–23]. Unlike fungal diversity, bacterial richness in this study population was not associated with vitamin A supplementation, but the bacterial community compositions trended toward significant dissimilarity [17]. There were no substantial differences in fungal alpha diversity between age groups; however, the fungal community compositions were shown to differ significantly between the younger (< 2 years) and older (4 to < 5 years) age groups, mostly attributed to the shift in the dominant genera, *Candida* and *Saccharomyces*. This shift may be ascribed to dietary changes, as older children are exposed to a larger variety of food and more yeast-containing food, like bread.

Comparable to the observations made in adults by the Human Microbiome Project, fungal gut diversity was much lower than bacterial diversity in the same participants [10]. A common theory for the relatively lower fungal gut diversity is that the gut environment is not conducive to the growth of most fungal taxa. Resident fungi must be able to grow at 37 °C to colonize the gut; therefore, many of the detected fungi are considered transient and are introduced through diet and inhalation [1, 24]. In fact, it has been suggested that the predominant “true” gut fungi are composed mainly of species from the genus *Candida*, and that other commonly identified fungi such as *Saccharomyces cerevisiae* and *Cladosporium* may only be present due to their abundance in food and drink, and the environment, respectively [25]. David and colleagues found that *Candida*, *Debaryomyces*, *Penicillium* and *Scopulariopsis* in the human gut were foodborne fungi associated with an animal-based diet [26]. In early life, some transient species such as *Malassezia* may be introduced through breastfeeding, but are later overtaken by members of the order Saccharomycetales [3].

In the present study, significant bacterial-fungal correlations were not very strong, and both positive and negative correlations were observed, which is different to what has been reported for healthy adults where positive correlations were more common [19, 27]. The strongest of the observed correlations was the co-occurrence of *Candida albicans* and *Ruminococcus gnavus* group. Similar correlations between *Candida* and *Ruminococcus* have been described in the gut [28]. The negative correlations previously found between *Penicillium* and *Faecalibacterium*, and *Saccharomyces* and the family Lachnospiraceae in the healthy adult gut [8] were not replicated in this study. Several fungi also displayed positive correlations with the family Enterobacteriaceae in this study; this correlation has recently been attributed to cooperation between these bacteria and fungi to facilitate colonisation [29]. Overall, fungal-fungal and bacterial-fungal correlations were weaker and less common than bacterial-bacterial correlations in the present study, but there is not enough evidence to determine whether this is a common trend in the human gut.

Despite extensive data collection efforts, the present study was limited by incomplete data for several clinical and epidemiological factors, and the lack of comprehensive dietary history for the participants. Another limitation of our study was the use of only the ITS1 region, which has a large variation in read length. While most fungi have short ITS1 regions (< 260 bp), some of the genera detected commonly in humans, such as *Saccharomyces*, have long regions that require longer paired-end sequencing reads (2 × 300 bp or more). Shorter regions could also potentially be over-amplified during the sequencing library preparation. An advantage of using ITS1 over ITS2 is the fact that ITS2 may be biased toward amplifying the Ascomycota, which are already extremely dominant in the gut [30]. ITS-based studies are also hampered by copy-number variation, which limits the ability to accurately quantify the abundance of species. We were not able to identify the core gut mycobiota due to the uncertainty of the colonisation potential of the detected fungi, and the low abundance of fungi in the gut. Despite these shortcomings, it was possible to assign taxonomy to at least the genus level in most cases, which could be attributed to the highly variable nature and subsequent discriminatory power of the ITS1 region.

## Conclusions

This study provides insight into the gut mycobiota of young African children and shows that the dominant taxa in this population are similar to those reported in other settings. Only age, sex and vitamin A supplementation were shown to be associated with the fungal diversity and composition. The connection between the microbiota, vitamin A supplementation, and growth and immunity warrants exploration, especially in populations at risk for micronutrient deficiencies. The low abundance of most of the detected fungal taxa and the lack of true gut colonising species among fungi remains an issue for mycobiota studies, due to the resultant difficulties in establishing the core mycobiota and determining which fungal taxa are truly affected by clinical and environmental factors. Functional studies are also necessary to explain the role of the mycobiota and the correlations between bacteria and fungi in human health.

## Methods

### Study design

This was a sub-study of the ongoing phase III, cluster randomised, double-blinded, placebo-controlled tuberculosis child multidrug-resistant preventive therapy (TB-CHAMP) trial (http://www.isrctn.com/ISRCTN92634082). TB-CHAMP aims to assess the efficacy and safety of levofloxacin as preventive therapy in children exposed to multidrug-resistant (MDR) tuberculosis (TB) within the household. This sub-study was a continuation of previous work that explored the bacterial gut microbiota of the children at their baseline visit, before randomisation into the treatment or placebo groups [17].

#### Population and participant eligibility

The study population has been described previously [17]. Briefly, children from the Cape Town metropolitan area under the age of 5 years who were residing in the same household as an enrolled adult index case (≥ 18 years of age) with bacteriologically confirmed pulmonary MDR-TB were eligible. The following were considered exclusion criteria: TB treatment in the previous 12 months; isoniazid or any fluoroquinolone treatment for ≥ 14 days at screening and known exposure to an isoniazid-susceptible TB index case.

#### Sample and participant data collection

One stool sample per child was collected without preservative in 25 mL faecal containers with spoons (Lasec, South Africa) at the baseline visit. Samples were transported to the laboratory, where they were homogenised and stored at -80ºC. A pilot study previously determined that the storage and transport conditions in this setting did not significantly affect microbiota composition, including fungi [31]. Demographic, clinical, and environmental data were collected during this visit using physical examinations, case report forms and lifestyle questionnaires. This included, but was not limited to, age, sex, HIV status, medical history (including mode of birth, breastfeeding habits, antibiotic use, deworming, and hospitalisation), anthropometric measurements, and exposure to environmental factors such as cigarette smoke, indoor cooking fires, pets, and day-care. Drinking water supply, ablution type and housing structure type were also recorded as a surrogate for socio-economic status. For age-related analyses, participants were grouped by year of life: A (0 to < 1 years) (*n* = 24), B (1 to < 2 years) (*n* = 25), C (2 to < 3 years) (*n* = 19), D (3 to < 4 years) (*n* = 24), and E (4 to < 5 years) (*n* = 23).

### DNA extraction and sequencing

DNA was extracted as part of a previous study investigating the bacterial gut microbiota of the study participants [17]. Briefly, the QIAamp PowerFecal DNA Isolation Kit (Qiagen, Germany), which includes a bead-beating step, was used according to the manufacturer’s instructions and stored at -20 °C. Sequencing was performed at the Centre for Proteomic and Genomic Research (CPGR) in Cape Town, on the Illumina MiSeq platform. Targeted sequencing was performed using the Illumina Fungal Metagenomic Demonstrated Sequencing Protocol and internal transcribed spacer 1 (ITS1) primer pool [32, 33] (Table 1). The amplicon PCR was adapted by increasing the number of cycles to 35 and the forward and reverse primer pools were added at concentrations of 2 μM and 1 μM, respectively. The MiSeq Reagent v3 Kit (600 cycles) was used to generate sequencing libraries for 2 × 200 bp paired-end read sequencing. Sequencing libraries were spiked with 10% of a 5 pM PhiX sequencing control, and the ZymoBIOMICS Microbial Community DNA standard (Zymo Research, USA) and batched negative extraction controls were included in the run.

**Table 1:** ITS1 primer pool used for Illumina sequencing

Primer pool	Sequence (5’ – 3’)	Reference
Forward		
ITS_fwd_1a	CTTGGTCATTTAGAGGAAGTAA	30
ITS_fwd_2	CTCGGTCATTTAGAGGAAGTAA	32
ITS_fwd_3	CTTGGTCATTTAGAGGAACTAA	32
ITS_fwd_4	CCCGGTCATTTAGAGGAAGTAA	32
ITS_fwd_5	CTAGGCTATTTAGAGGAAGTAA	32
ITS_fwd_6	CTTAGTTATTTAGAGGAAGTAA	32
ITS_fwd_7	CTACGTCATTTAGAGGAAGTAA	32
ITS_fwd_8	CTTGGTCATTTAGAGGTCGTAA	32
Reverse		
ITS_rev_1b	GCTGCGTTCTTCATCGATGC	30
ITS_rev_2	GCTGCGTTCTTCATCGATGG	32
ITS_rev_3	GCTACGTTCTTCATCGATGC	32
ITS_rev_4	GCTGCGTTCTTCATCGATGT	32
ITS_rev_5	ACTGTGTTCTTCATCGATGT	32
ITS_rev_6	GCTGCGTTCTTCATCGTTGC	32
ITS_rev_7	GCGTTCTTCATCGATGC	32

### Microbiome analysis and statistical testing

#### Sequence analysis and taxonomic classification

Sequence analysis was performed using the Quantitative Insights Into Microbial Ecology (QIIME2 2020.8) bioinformatics platform [34] in conjunction with other bioinformatics packages described below, on the Stellenbosch University high performance computing cluster (HPC) 2 (http://www.sun.ac.za/hpc). ITS1 regions were extracted using ITSxpress [35] in order to remove read-through sequences from shorter ITS1 amplicons and improve read merging in paired-end sequences. The dada2 plug-in [36] was used to perform read error correction, quality filtering and chimaera removal, and feature tables were generated using amplicon sequence variants (ASVs). Taxonomy was assigned to the sequence features outputted from dada2, using a classifier trained with the feature-classifier plug-in [37, 38] on the UNITE fungal database version 8.2 (https://doi.org/10.15156/BIO/786380035). Forward read analysis was performed as described for paired-end reads, except for the ITSxpress step, which was replaced with adapter- and read-through trimming using the cutadapt plug-in [39].

#### Microbial community diversity

Diversity analyses were performed at the feature level, and rarefaction, supported by Good’s coverage, was performed prior to analysis. The evenness and richness in participant samples (alpha diversity) was demonstrated using the Shannon’s H metric [40] and observed features (OF), and the significance between groups, based on clinical, demographic, and socio-economic factors, was determined using Kruskal–Wallis pairwise tests [41]. Spearman correlation analysis was performed for numerical data [42]. The Shannon alpha diversity of fungal communities was compared to that of bacterial communities in the same participant samples, investigated/described previously [17]. Microbial community dissimilarity (beta diversity) was determined with principal coordinate analysis (PCoA) based on the Bray–Curtis dissimilarity metric [43] and dissimilarity based on presence/absence was evaluated with Jaccard distance [44]. Statistical differences were determined by PERMANOVA [45]. For Bray–Curtis similarity, the adonis plug-in [46] was also implemented to report R^2^ values to show the strength of associations between tested factors. Benjamini–Hochberg False Discovery Rate (BH-FDR) multiple test correction was performed where appropriate and the threshold for significance was 0.05 for all p values and adjusted *p* values (q values) [47].

#### Differential abundance

Differentially abundant features for factors such as age, medication use, method of birth, breastfeeding, and exposure to pets and smoke were investigated at genus level using Analysis of composition of microbiomes (ANCOM) [48]. ANCOM performs pairwise testing and generates a test statistic (W), with a significance threshold, which shows how many times a hypothesis could be rejected for a specific genus. As recommended, only features present ≥ 20 times overall and in at least 25% of the samples were used for ANCOM analyses, to avoid noise from less abundant features.

#### Correlations between bacterial and fungal microbiota

FastSpar, an implementation of the Sparse Correlations for Compositional data (SparCC) method, was used to investigate the correlations between bacterial and fungal species in the gut environment and to test the significance of the correlations [49, 50]. Samples with less than 500 feature counts were excluded, and only features that were present in more than one sample and made up at least 0.5% of the overall abundance were included in correlation analysis. The correlations were plotted in R using corrplot version 0.89 [51]. FastSpar produces p values following a permutation-based approach and bootstrapping; these p values and raw correlation values can be found in Supplementary Tables S6 and S7.

## Supplementary Information

**Additional file 1. **

## Data Availability

The sequencing data analysed during the current study are available through the NCBI Sequence Read Archive under BioProject PRJNA692358.
