# Supplementary material for: Fungal diversity in the gut microbiome of young South African children
Source: BMC Microbiol. 2022 Aug 17;22:201. doi: 10.1186/s12866-022-02615-w (PMC9387017; doi:10.1186/s12866-022-02615-w)
Supplement: Supplementary file 1 — Additional file 1. [file 12866_2022_2615_MOESM1_ESM.docx]

# Supplementary Material for:

# Fungal diversity in the gut microbiome of young South African children

K Nel Van Zyl^1^, AC Whitelaw^1,2,3^, AC Hesseling^4^, JA Seddon^4,5^, A-M Demers^4,6^, and M Newton-Foot^1,2^

1. Division of Medical Microbiology, Department of Pathology, Stellenbosch University, South Africa
2. National Health Laboratory Service, Tygerberg Hospital, Cape Town, South Africa
3. African Microbiome Institute, Stellenbosch University, South Africa
4. Desmond Tutu TB Centre, Department of Paediatrics and Child Health, Stellenbosch University, South Africa
5. Department of Infectious Diseases, Imperial College London, United Kingdom
6. Service de microbiologie, Département clinique de médecine de laboratoire, Centre Hospitalier Universitaire Sainte-Justine, Montréal, Canada

# Results

## Comparison between paired-end and forward read only analysis

The ZymoBIOMICS Microbial Community DNA standard (Zymo Research, USA) contains two fungal species in equal proportion, *Cryptococcus* *neoformans* and *Saccharomyces* *cerevisiae*, and was used to evaluate the performance of the ITS1 sequencing. Following taxonomic assignment, it was clear that the 2 x 200b paired-end reads used in this study could not resolve the ITS1 region in *Saccharomyces* spp. (480 bp), whereas forward read analysis detected both genera which were present in the mock community standard (Fig S1).


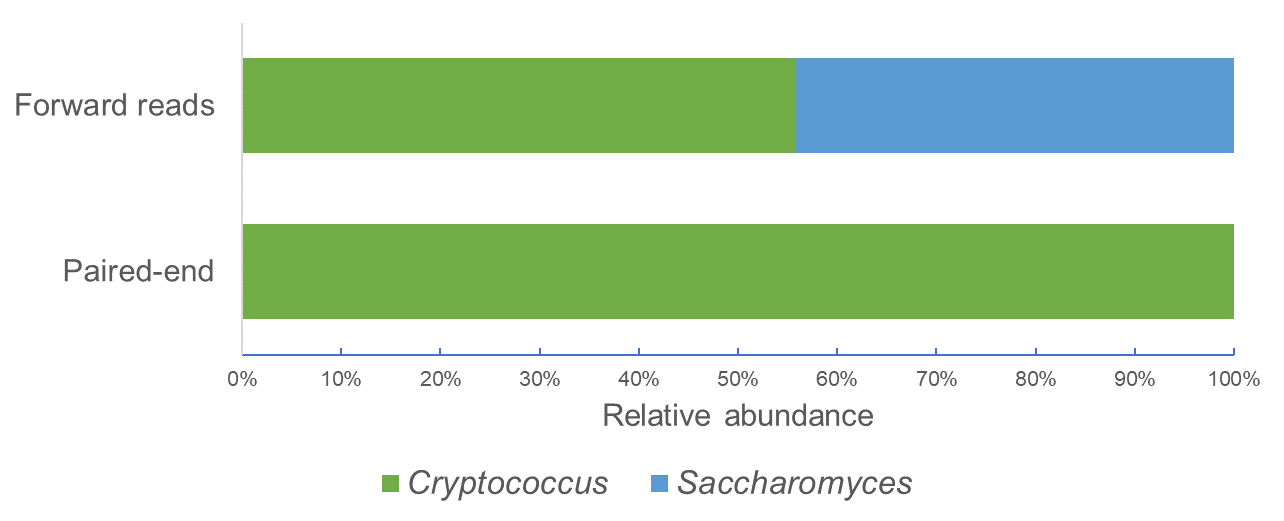


Fig S1. Comparison of the taxonomy assignment of the mock community control using merged pair-end reads versus forward only reads.

While most of the detected fungi could be assigned to genus level, and 6 of the top 10 detected taxa were shared between the two sequence analysis approaches (Table S1), forward read analysis detected more taxa than paired-end analysis. Further, *Saccharomyces* spp. was shown to be present in most of the population and the forward reads were therefore used for all subsequent abundance and diversity analyses.

**Supplementary Table S1. Percentage of fungal taxa detected in samples in children <5 years.** The top 10 taxa are shown and shared genera have been highlighted in bold font. Taxa unassigned or unidentified at phylum level were filtered prior to analysis.

| **Paired-end** | | | **Forward** **reads** | | |
| --- | --- | --- | --- | --- | --- |
|  | **Taxon** | **Detection rate** |  | **Taxon** | **Detection rate** |
| 1 | ***Candida*** | 96% | 1 | ***Candida*** | 100% |
| 2 | ***Rhodotorula*** | 50% |  | *Saccharomyces* |  |
| 3 | ***Malassezia*** | 49% | 3 | Unassigned Ascomycota | 96% |
| 4 | ***Cladosporium*** | 42% | 4 | ***Aspergillus*** | 77% |
| 5 | *Alternaria* | 37% | 5 | ***Malassezia*** | 75% |
| 6 | *Wallemia* | 36% | 6 | ***Cyberlindnera*** | 73% |
| 7 | ***Aspergillus*** | 34% | 7 | ***Rhodotorula*** | 65% |
| 8 | Unassigned Saccharomycetales | 33% | 8 | *Penicillium* | 62% |
| 9 | *Issatchenkia* | 30% | 9 | *Metschnikowia* | 60% |
| 10 | ***Cyberlindnera*** | 25% | 10 | ***Cladosporium*** | 58% |
|  | *Debaromyces* |  |  |  |  |
|  | *Kluyveromyces* |  |  |  |  |

**Supplementary Table S2. Differences in fungal alpha diversity between groups based on Shannon’s H metric and observed features (OF).**

|  | | **Shannon’s H** | | | **Observed features (OF)** | | |
| --- | --- | --- | --- | --- | --- | --- | --- |
| Category | | **Kruskal-Wallis test statistic** | **p-value** | **Corrected  p-value** | **Kruskal-Wallis test statistic** | **p-value** | **Corrected  p-value** |
| Demographics | | | | | | | |
| Sex (Male vs Female) | | 8.690 | **0.003** | - | 3.509 | 0.061 | - |
| Maternal HIV status (Positive vs Negative) | | 0.986 | 0.321 | - | 1.889 | 0.169 | - |
| Method of birth (NVD vs C/S) | | 0.504 | 0.478 |  | 5.331 | **0.021** |  |
| Group A only  (0 to <1 years) | **Method of birth (NVD vs C/S)** | 0.027 | 0.870 | - | 0.002 | 0.967 | - |
|  | **Premature birth (<37 weeks gestation)** | 1.000 | 0.317 | - | 0.090 | 0.764 | - |
|  | **Breastfeeding (first six months of life)** | 2.492 | 0.114 | - | 1.851 | 0.174 | - |
|  | **Exclusive breastfeeding** | 1.500 | 0.221 | - | 3.533 | 0.060 | - |
| Groups C+D+E  (2 to <5 years) | **Day-care exposure** | 1.387 | 0.239 | - | 0.465 | 0.495 | - |
| Clinical factors | | | | | | | |
| Antibiotic receipt (<2 weeks from baseline) | | 3.317 | 0.069 | - | 4.646 | **0.031** | - |
| Antibiotic receipt (<6 months from baseline) | | 0.088 | 0.767 | - | 0.003 | 0.955 | - |
| Hospital admission (<6 months from baseline) | | 0.485 | 0.486 | - | 0.055 | 0.814 | - |
| Hospital admission (first 6 months of life) | | 0.016 | 0.901 | - | 0.296 | 0.586 | - |
| Visit to traditional healer (<6 months from baseline) | | 0.109 | 0.741 | - | 2.100 | 0.147 | - |
| Dewormed (<6 months from baseline) | | 0.852 | 0.356 | - | 1.792 | 0.181 | - |
| Vitamin A supplementation (<6 months from baseline, children >6 months) | | 3.935 | **0.047** | - | 0.292 | 0.589 | - |
| Environmental factors | | | | | | | |
| Cigarette smoke exposure from mother and/or household | | 0.469 | 0.493 | - | 0.174 | 0.677 | - |
| Indoor cooking fire exposure | | 0.243 | 0.622 | - | 0.548 | 0.459 | - |
| Pets (cats/dogs) in household | | 2.307 | 0.129 | - | 2.480 | 0.115 | - |
| Sample storage  Fridge vs On ice packs Fridge vs Room temp On ice vs Room temp | | 0.109 1.007 1.157 | 0.741 0.316 0.282 | 0.741 0.474 0.474 | 2.003 0.002 0.859 | 0.157 0.960 0.354 | 0.471 0.960 0.531 |
| Socio-economic factors | | | | | | | |
| Household structure Brick structure vs Shed/Prefab house  Brick structure vs Shack Shed/Prefab house vs Shack | | 0.430  0.616  0.117 | 0.512  0.432  0.732 | 0.732  0.732  0.732 | 1.432 0.070 1.463 | 0.232 0.791 0.227 | 0.347 0.791 0.347 |
| Ablution type  Bucket system vs Exclusive flush toilet (in house/outside)  Bucket system vs Shared flush toilet  Bucket system vs Pit/VIP latrine  Exclusive flush toilet (in house/outside) vs Shared flush toilet  Exclusive flush toilet (in house/outside) vs Pit/VIP latrine  Shared flush toilet vs Pit/VIP latrine | | 3.736  4.149  1.484  0.058  0.138  0.050 | 0.053  0.042  0.223  0.810  0.710  0.823 | 0.160  0.160  0.446  0.823  0.823  0.823 | 2.575  2.780  1.130  0.292  0.120  0.451 | 0.109  0.095  0.288  0.589  0.730  0.502 | 0.326  0.326  0.576  0.707  0.730  0.707 |
| Drinking water supply  Piped water from a public shared tap vs in residence Piped water from a public shared tap vs exclusive tap outside Piped water in the residence vs exclusive tap outside | | 0.806 1.778  1.025 | 0.369 0.182  0.311 | 0.369 0.369  0.369 | 0.565 0.445  0.011 | 0.452 0.505  0.918 | 0.757 0.757  0.918 |

For factors with >2 groups, the corrected p-values have been given (Benjamini-Hochberg False Discovery Rate (BH-FDR) multiple test correction). Nineteen samples were excluded following rarefaction and samples with no data for a particular factor were excluded from that analysis.

VIP latrine= Ventilated improved pit latrine

**Supplementary Table S3. Spearman correlation for fungal diversity associations in numerical data.**

| Category | | **Spearman test statistic** | **p-value** |
| --- | --- | --- | --- |
| Group A:  (0 to <1 years) | Duration of breastfeeding (months) | -0.062 | 0.856 |
|  | Duration of exclusive breastfeeding (months) | 0.248 | 0.521 |
|  | Age solid food was introduced (months) | 0.096 | 0.725 |
| Groups C+D+E:  (2 to <5 years) | Age day-care was started (months) | -0.217 | 0.319 |
|  | Size of day-care group | -0.125 | 0.569 |
|  | Hours a day spent in day-care | -0.076 | 0.736 |

Nineteen samples were excluded following rarefaction and samples with no data for a particular factor were excluded from that analysis.

**Supplementary Table S4. Differences between fungal and bacterial alpha diversity in different age groups, based on Shannon’s H metric.**

| Age groups | **Kruskal-Wallis test statistic** | **p-value** | **Corrected  p-value** |
| --- | --- | --- | --- |
| FUNGI | | | |
| A vs B | 0.18 | 0.67 | 0.85 |
| A vs C | 0.04 | 0.85 | 0.85 |
| A vs D | 0.07 | 0.79 | 0.85 |
| A vs E | 1.37 | 0.24 | 0.85 |
| B vs C | 0.11 | 0.74 | 0.85 |
| B vs D | 0.37 | 0.54 | 0.85 |
| B vs E | 0.98 | 0.32 | 0.85 |
| C vs D | 0.39 | 0.53 | 0.85 |
| C vs E | 0.74 | 0.39 | 0.85 |
| D vs E | 1.60 | 0.21 | 0.85 |
| BACTERIA | | | |
| A vs B | 11.77 | <0.01 | <0.01 |
| A vs C | 21.66 | <0.01 | <0.01 |
| A vs D | 16.37 | <0.01 | <0.01 |
| A vs E | 27.29 | <0.01 | <0.01 |
| B vs C | 9.50 | <0.01 | <0.01 |
| B vs D | 3.37 | 0.07 | 0.09 |
| B vs E | 16.40 | <0.01 | <0.01 |
| C vs D | 0.96 | 0.33 | 0.33 |
| C vs E | 1.45 | 0.23 | 0.25 |
| D vs E | 3.23 | 0.07 | 0.09 |

For factors with >2 groups, the corrected p-values have been given (Benjamini-Hochberg False Discovery Rate (BH-FDR) multiple test correction).

Nineteen samples from the fungal analysis and five from the bacterial analysis were excluded following rarefaction.

A: 0 to <1 years

B: 1 to <2 years

C: 2 to <3 years

D: 3 to <4 years

E: 4 to <5 years

**Supplementary Table S5. Bray-Curtis p-values and R^2^ values as reported by the adonis plug-in on QIIME2.**

| Age groups | **p(q)-value** | **R^2^** |
| --- | --- | --- |
| A vs B | 0.272 | 0.027916 |
| A vs C | 0.117 | 0.043492 |
| A vs D | 0.005 | 0.075284 |
| A vs E | 0.001 | 0.097744 |
| B vs C | 0.052 | 0.045165 |
| B vs D | 0.003 | 0.076942 |
| B vs E | 0.002 | 0.082932 |
| C vs D | 0.148 | 0.041113 |
| C vs E | 0.092 | 0.046886 |
| D vs E | 0.495 | 0.025819 |

**Supplementary Table S6. Correlation values for bacterial and fungal taxa.** The top ten positive correlations are highlighted blue and the top ten negative correlations are highlighted in red.

**Supplementary Table S7. P values for the correlation between bacterial and fungal taxa.** The significant p values (<0.05) have been highlighted in green.
